# Biotechnological applications of mobile group II introns and their reverse transcriptases: gene targeting, RNA-seq, and non-coding RNA analysis

**DOI:** 10.1186/1759-8753-5-2

**Published:** 2014-01-13

**Authors:** Peter J Enyeart, Georg Mohr, Andrew D Ellington, Alan M Lambowitz

**Affiliations:** 1 Departments of Molecular Biosciences and Chemistry, Institute for Cellular and Molecular Biology, The University of Texas at Austin, Austin, TX 78712, USA

**Keywords:** Genome engineering, Metabolic engineering, Next-generation RNA sequencing, Ribozyme, Synthetic biology, Systems biology, Targetron

## Abstract

Mobile group II introns are bacterial retrotransposons that combine the activities of an autocatalytic intron RNA (a ribozyme) and an intron-encoded reverse transcriptase to insert site-specifically into DNA. They recognize DNA target sites largely by base pairing of sequences within the intron RNA and achieve high DNA target specificity by using the ribozyme active site to couple correct base pairing to RNA-catalyzed intron integration. Algorithms have been developed to program the DNA target site specificity of several mobile group II introns, allowing them to be made into ‘targetrons.’ Targetrons function for gene targeting in a wide variety of bacteria and typically integrate at efficiencies high enough to be screened easily by colony PCR, without the need for selectable markers. Targetrons have found wide application in microbiological research, enabling gene targeting and genetic engineering of bacteria that had been intractable to other methods. Recently, a thermostable targetron has been developed for use in bacterial thermophiles, and new methods have been developed for using targetrons to position recombinase recognition sites, enabling large-scale genome-editing operations, such as deletions, inversions, insertions, and ‘cut-and-pastes’ (that is, translocation of large DNA segments), in a wide range of bacteria at high efficiency. Using targetrons in eukaryotes presents challenges due to the difficulties of nuclear localization and sub-optimal magnesium concentrations, although supplementation with magnesium can increase integration efficiency, and directed evolution is being employed to overcome these barriers. Finally, spurred by new methods for expressing group II intron reverse transcriptases that yield large amounts of highly active protein, thermostable group II intron reverse transcriptases from bacterial thermophiles are being used as research tools for a variety of applications, including qRT-PCR and next-generation RNA sequencing (RNA-seq). The high processivity and fidelity of group II intron reverse transcriptases along with their novel template-switching activity, which can directly link RNA-seq adaptor sequences to cDNAs during reverse transcription, open new approaches for RNA-seq and the identification and profiling of non-coding RNAs, with potentially wide applications in research and biotechnology.

## Review

### Introduction

Mobile group II introns are bacterial retrotransposons that perform a remarkable ribozyme-based, site-specific DNA integration reaction (‘retrohoming’) and encode an equally remarkable reverse transcriptase (RT), both of which have been harnessed for biotechnological applications [1-3]. Retrohoming occurs by a mechanism in which the group II intron RNA uses its ribozyme activity to insert directly into a DNA strand, where it is reverse transcribed by the intron-encoded RT (also referred to as the intron-encoded protein or IEP), yielding a cDNA copy of the intron that is integrated into the genome [4]. Because mobile group II introns recognize DNA target sequences largely by base pairing of sequence motifs within the intron RNA, they can be programmed to insert into desired DNA sites by simply modifying the intron sequences so as to base pair to the new target site. This feature allows mobile group II introns to be made into gene targeting vectors, or ‘targetrons’, which combine high DNA integration efficiency with readily programmable and reliable DNA target specificity [5-7]. Targetrons are widely used for genetic engineering of bacteria, and efforts continue to adapt them for function in eukaryotes.

Group II intron RTs function in retrohoming by synthesizing a full-length cDNA of the highly structured intron RNA with high processivity and fidelity [8-10], properties that are useful for biotechnological applications involving cDNA synthesis, such as qRT-PCR and next-generation RNA sequencing (RNA-seq). The RTs also have a novel template-switching activity that enables facile attachment of adaptor sequences containing primer-binding sites and barcodes to cDNAs. These properties, combined with the availability of naturally occurring thermostable group II intron RTs [11,12] open new approaches for RNA-seq and the profiling and discovery of miRNAs and other non-coding RNAs [10,13].

Here, we describe how the novel biochemical activities of mobile group II introns and their RTs, which were acquired during the evolution of group II introns as mobile genetic elements, have been adapted for biotechnological applications. We then review how group II intron-derived targetrons have been used for the genetic engineering of diverse bacteria, as well as recent advances in targetron technology. The latter include the development of a thermotargetron for gene targeting in thermophiles, methods for using targetrons to position recombinase recognition sites for large-scale genome rearrangements, and progress in developing targetrons for gene targeting in eukaryotes. Finally, we discuss the development of thermostable group II intron RTs from bacterial thermophiles as new tools for cDNA synthesis, with potentially wide applications in research and biotechnology.

### Mobile group II introns

Mobile group II introns are found in bacteria, archaea, and the mitochondrial and chloroplast DNAs of some eukaryotes, and are thought to be evolutionary ancestors of spliceosomal introns, the spliceosome, retrotransposons, and retroviruses in higher organisms [3,14,15]. They are especially prevalent and widespread in bacteria, with hundreds of bacterial group II introns having been identified by genome sequencing [16].

**Figure 1:**
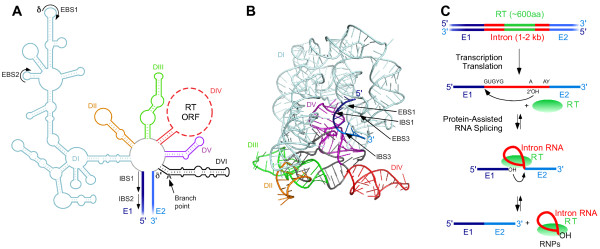
**Group II intron RNA structure and splicing mechanism. (A)** Group II intron RNA secondary structure. The example shown is the *Lactococcus lactis* Ll.LtrB group IIA intron. Intron RNA domains are different colors, and the 5’ and 3’ exons (E1 and E2, respectively) are thicker dark and light blue lines, respectively. The large ‘loop’ region of DIV, which encodes the group II intron RT, is shown as a dashed line and not drawn to scale. **(B)** Crystal structure of the *Oceanobacillus iheyensis* group IIC intron. The ribbon diagram of the intron’s structure was generated from Protein Data Bank file 3IGI [20] (http://www.pdb.org) with PyMol. Group II intron RNA domains are colored as in panel **A**. **(C)** Group II intron RNA splicing and reverse splicing. Double-stranded DNA is indicated by double lines and RNA as a single line. E1 and E2 are shown in dark and light blue, respectively; the intron and intron RNA are shown in red; and the intron-encoded RT is shown in green.

Mobile group II introns consist of a catalytically active intron RNA, which encodes an RT (Figure 1) [1-3,17]. Group II intron RNAs have a length of 400 to 800 nts, excluding the ORF encoding the RT [3]. They have little sequence similarity to each other, but fold into a conserved three-dimensional structure consisting of six interacting double helical domains (DI-DVI) (Figure 1A and B) [17-21].

The folded group II intron RNA contains an active site that uses specifically bound Mg^2+^ ions to catalyze RNA splicing via two sequential transesterification reactions that yield ligated exons and an excised intron lariat RNA, the same reaction mechanism used for the splicing of nuclear spliceosomal introns in eukaryotes (Figure 1C) [1]. Because the transesterification reactions used for splicing are reversible, the intron RNA can also catalyze reverse splicing of the intron into RNA or DNA sites containing the ligated exon sequence, with reverse splicing into DNA playing a key role in intron mobility. Both steps of reverse splicing (referred to as complete reverse splicing) result in the insertion of the excised intron RNA between the 5’ and 3’ exons, while the first step (referred to as partial reverse splicing) results in the attachment of the 3’ end of the intron RNA to the 5’ end of the downstream exon, leaving a strand break.

Some key regions of group II intron RNAs are DI, which contains the motifs that base pair with the DNA target site; DIV, which contains the ORF encoding the RT; DV, a metal-ion-binding domain that comprises most of the active site; and DVI, which contains the branch-point nucleotide [19]. Three subclasses of group II introns, denoted IIA, IIB, and IIC, have been distinguished by variations of the conserved RNA structure [3]. Crystal structures of a group IIC intron at different stages of reaction have been determined, providing insight into the nature of the active site and the mechanisms of RNA splicing and reverse splicing (Figure 1B) [19-21].

**Figure 2:**
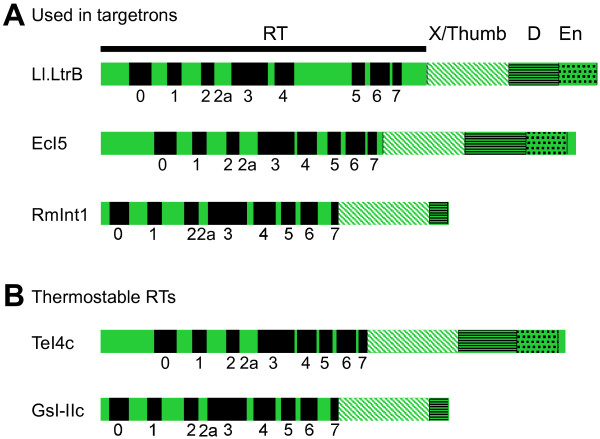
**Group II intron reverse transcriptases (RTs). (A)** Group II intron RTs encoded by the *L. lactis* Ll.LtrB, *E. coli* EcI5, and *Sinorhizobium meliloti* RmInt1 introns, which have been converted into targetrons. The Ll.LtrB RT is also referred to as the LtrA protein. **(B)** Thermostable group II intron RTs from bacterial thermophiles used for biotechnological applications involving cDNA synthesis, such as qRT-PCR, RNA-seq, and miRNA profiling. Group II intron domains are: RT, with conserved RT sequence blocks 1 to 7 found in all RTs (black rectangles) and additional conserved regions RT-0 and RT-2a also found in non-LTR-retrotransposon RTs [23]; X/thumb, white hatching; DNA binding (D), horizontal black lines; DNA endonuclease (En), black dots.

Group II intron RTs typically consist of 400 to 600 amino acids and contain a series of conserved motifs characteristic of retroviral and other RTs [3]. Figure 2 shows schematics of several group II intron RTs that are discussed in this review. Group II intron RTs contain conserved N-terminal RT and X domains, which correspond to the fingers/palm and thumb domains of retroviral RTs, respectively. In addition to reverse transcription, the RT and X domains bind specifically to the intron RNA to stabilize the active ribozyme structure for RNA splicing and reverse splicing (referred to as ‘maturase’ activity, an example of protein-assisted RNA catalysis). Group II intron RTs lack an RNase H domain, but typically have C-terminal DNA-binding (D) and DNA endonuclease (En) domains that interact with DNA target sites during retrohoming. Some IEPs, such as that encoded by RmInt1 from *Sinorhizobium meliloti*[22] (Figure 2A), lack the En domain. Notably, the RT and thumb domains of group II intron RTs are larger than those of retroviral RTs and contain an N-terminal extension and several distinctive ‘insertions’ between the conserved RT sequence blocks [23]. The larger RT and thumb domains may enable more extensive interactions with RNA templates and thus contribute to the high processivity of group II intron RTs (see below).

### Group II intron retrohoming

**Figure 3:**
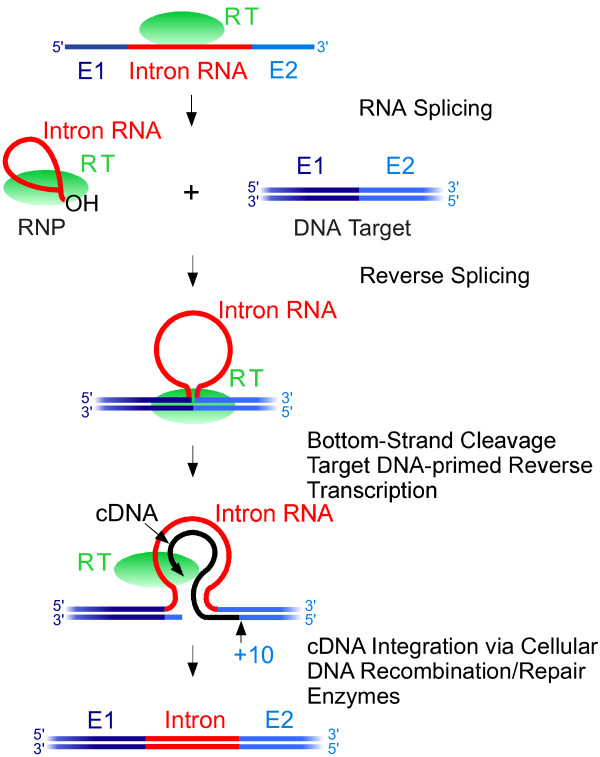
**Group II intron retrohoming.** In the first step, the reverse transcriptase (RT) binds to the intron in a larger initial transcript of a gene and promotes RNA splicing, resulting in a ribonucleoprotein (RNP) complex that contains the excised intron lariat RNA and the tightly bound RT. RNPs recognize DNA target sites by using both the RT and base pairing of the intron RNA and then promote reverse splicing of the intron RNA into the top strand of the double-stranded DNA. After reverse splicing, the bottom DNA strand is cleaved by the En domain of the RT, and the 3’ end generated at the cleavage site is used as a primer for target DNA-primed reverse transcription of the inserted intron RNA. The resulting intron cDNA (black) is integrated into the host genome by cellular DNA recombination or repair mechanisms.

Group II intron retrohoming has been reviewed in detail elsewhere [3,4], and here we describe only the major steps and variations pertinent to the mechanism of gene targeting. As shown in Figure 3, retrohoming starts with the group II intron splicing out of a larger RNA molecule, typically a transcript of the gene in which the group II intron is inserted. Splicing is accomplished via folding of the intron RNA into a catalytic structure, with help of the RT, which binds the intron RNA and stabilizes the active RNA tertiary structure. As discussed above, splicing occurs via two transesterification reactions that yield ligated exons and an excised intron lariat. After splicing, the RT remains tightly bound to the excised intron lariat RNA in a ribonucleoprotein (RNP) complex that initiates retrohoming by recognizing DNA target sequences by a combination of site-specific binding of the RT and base pairing of sequence motifs in the intron RNA, described in detailed below. The intron RNA then integrates directly into the DNA target site by full reverse splicing (see above), while the endonuclease activity of the RT cuts the opposite DNA strand slightly downstream of the insertion site, leaving an overhang with a cleaved 3’ end that is used as a primer for synthesis of a cDNA copy of the inserted intron RNA by the RT [24-26]. Introns encoding RTs lacking the endonuclease activity retrohome by using nascent lagging or leading DNA strands at DNA replication forks as primers for reverse transcription [27-29].

The cDNA copy of the reverse-spliced intron RNA is integrated into the host genome by common cellular DNA recombination or repair mechanisms, a feature that contributes to the wide host range of group II introns. Recent findings have further elucidated late steps in group II intron integration in *Escherichia coli*, in which a cellular RNase H degrades the intron RNA, and replication restart proteins then recruit the host replicative DNA polymerase, which synthesizes DNA corresponding to the sense strand of the intron [30]. Host nucleases trim DNA overhangs, and ligases repair remaining nicks [31].

Some group II introns splice via hydrolysis rather than branching and thus excise a linear rather than a lariat intron RNA [32,33]. During retrohoming, linear group II intron RNAs can carry out only the first step of reverse splicing, attaching the 3’ end of the linear intron to the downstream DNA exon, which, combined with En cleavage of the opposite strand, yields a double-strand break that can be repaired by homologous recombination with exogenous DNA [34]. This double-strand break-stimulated recombination provides an alternative gene targeting mechanism for group II introns, analogous to that used by Zn-finger nucleases, TALENs, and CRISPR-based systems [35]. In some hosts, the linear group II intron RNA inserted at a target site is reverse-transcribed to yield a cDNA that can be integrated into the genome by non-homologous end joining [36,37].

### DNA-target site recognition

**Figure 4:**
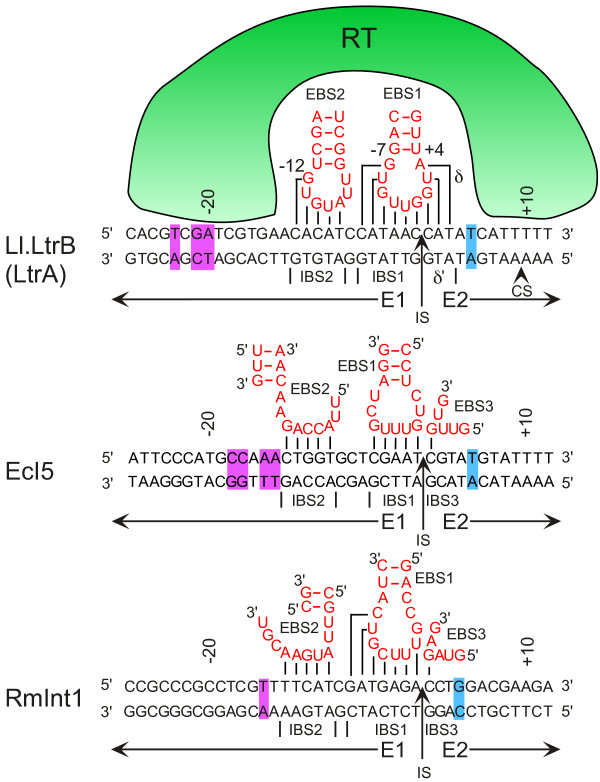
**DNA target site recognition by mobile group II introns.** The figure shows DNA target-site interactions for the Ll.LtrB group IIA intron and the EcI5 and RmInt1 group IIB introns used as targetrons. Portions of the intron RNA involved in the EBS1-IBS1, EBS2-IBS2, and δ − δ’ or EBS3-IBS3 base-pairing interactions with the DNA target site are shown in red. Purple and blue highlights indicate base-pairs in the 5’ and 3’ exons (E1 and E2, respectively) that are important for DNA targeting and recognized by the reverse transcriptase (RT). CS, bottom-strand cleavage site; IS, intron-insertion site.

The key to using group II introns for gene targeting is their mode of DNA target site recognition. Group II intron RNPs recognize DNA target sequences by using both the RT and base pairing of the intron RNA, with the latter contributing most of the DNA target specificity [5,38]. Group IIA, IIB, and IIC introns differ somewhat in how they recognize DNA target sites, and these differences impact design and performance in the biotechnological context. The major target site interactions for the *Lactococcus lactis* Ll.LtrB intron, the most widely used for gene targeting, and several other group II introns used as targetrons are illustrated in Figure 4.

In group IIA introns, like the Ll.LtrB intron, the intron RNA contains three sequence motifs in DI that recognize DNA target sites by base pairing. These are denoted EBS1, EBS2, and δ, and they base pair to complementary sequences in the DNA target site denoted IBS1, IBS2, and δ’ (where EBS stands for ‘exon-binding site’ and IBS stands for ‘intron-binding site’; these same interactions also occur upon splicing out of a larger RNA molecule). The Ll.LtrB RT (denoted LtrA protein) recognizes nucleotides both upstream and downstream of the IBS/δ’ sequences (colored purple and blue, respectively, in Figure 4). Binding of the RT promotes DNA melting [39], enabling the intron RNA to base pair to the DNA target sequence, and DNA bending, which positions the target DNA properly for cleavage and priming of reverse transcription [40].

Group IIB introns, like EcI5 and RmInt1, also contain three sequence elements that recognize the DNA target site by base pairing. Specifically, EBS1, EBS2, and EBS3 base pair to corresponding IBS sequences in the target. The EBS3 sequence is located in a different part of the DI structure than the corresponding δ sequence in group IIA introns [41]. The RT again recognizes flanking sequences. In EcI5, a relatively well-studied example of this class [42], the RT recognizes a similar number of residues as the RT of Ll.LtrB, although the identities and locations of these residues differ. RmInt1, whose RT lacks the En domain, inserts into the single-stranded DNA formed during replication and thus has no requirement for DNA melting [28]. The RmInt1 RT recognizes only two critical nucleotide residues, but additional sequences may contribute [43].

Group IIC introns recognize short IBS1 and IBS3 sequences. A DNA hairpin, such as those found in gene terminators or phage attachment sites, is also a key recognition determinant and seems to take the place of the IBS2 sequence for these introns, although the mechanism of recognition is as yet unknown [44-46]. Group IIC introns can thus integrate into multiple sites, and specificity is limited.

Group IIA and IIB introns have high DNA-target specificity and integrate only rarely into ectopic sites (for example, retrotransposition of the Ll.LtrB intron into ectopic sites in the *E. coli* chromosome occurs at a frequency of 0.1 to 30 × 10^-6^) [3,47]. This high specificity reflects, in part, the fact that group II introns use both the RT and base pairing of the intron RNA to recognize their DNA target sequences, with the RTs of the Ll.LtrB and EcI5 introns most stringently recognizing 4 to 5 nts and intron RNA base pairing extending over 11 to 14 nts spanning the intron-insertion site. Additionally, because the heteroduplex between the intron RNA and DNA target strand must bind to the intron RNA’s active site for reverse splicing, mismatches in base pairing strongly affect the k_cat_ as well as K_m_ of the targeting reaction, providing greater discrimination against mispairings than can be obtained by binding affinity alone [48].

This intertwining of DNA target binding and catalysis differs from CRISPR-based systems, which have been used in bacteria and eukaryotes and also rely on base-pairing between RNA and DNA to provide specificity [49-55]. CRISPR systems use a guide RNA bound by a protein endonuclease (Cas9 being the canonical example) and can in theory target any stretch of twenty base pairs that is followed by a specific ‘protospacer adjacent motif’ (PAM), which in currently utilized systems is a stretch of two to five nts recognized by the endonuclease. However, the guide RNA does not play a catalytic role and thus specificity appears to be governed solely by its binding affinity to the DNA target site, with the protein endonuclease cutting anytime the RNA/protein complex tarries long enough at a given site. Indeed, concerns have been raised about the high off-targeting rate of these systems, with off-target sites having up to five mismatches found to be targeted at efficiencies similar to the intended site [56]. A further limitation for wide use in bacteria is that, unlike group II introns, CRISPR-based systems function only to introduce a double-strand break, and integration of exogenous DNA at the break site is dependent upon homologous recombination at a higher efficiency than is found in most bacterial species [53].

### Targetrons

Because mobile group II introns recognize their DNA target sites by a combination of base-pairing interactions and site-specific binding of the RT, the target site recognized by the RNP can be modified by finding other sites compatible with RT recognition and then changing the EBS/δ sequences of the intron as necessary to match the new site [5]. Such retargeted mobile group II introns are called ‘targetrons.’ Group II introns that have been made into targetrons include both group IIA introns (Ll.LtrB [7]) and group IIB introns (EcI5 [42] and RmInt1 [57]). Group IIC introns are less appealing as candidates for retargeting because they recognize hairpin structures via as yet unknown mechanisms. The Ll.LtrB targetron is commercially available through Sigma-Aldrich, and both the Ll.LtrB and EcI5 targetrons are available through Targetronics.

Although group II introns can and have been retargeted by the method mentioned above, in which the closest match to the native recognition site in a sequence to be targeted is identified, and the base-pairing sequences of the intron are modified to accommodate discrepancies, the rules by which introns recognize their target sites are actually more complex. For instance, the RT recognizes different residues at the target site with different stringencies, and none of these recognition events are absolutely required for retrohoming to occur [5,58,59]. If only the wild-type recognition sequence is used, then new targeting sites may be hard to come by, but knowing which bases can be varied and how is not a simple matter. The EBS/δ sequences may also differ in the stringency of required base-pairing interactions at different positions. Algorithms have therefore been developed for retargeting the Ll.LtrB [7] and EcI5 [42] introns. These algorithms were developed by examining libraries of inserted mobile group II introns with randomized base-pairing motifs for the most frequently conserved residues and base-pairing interactions, and using these frequencies to generate weighting schemes for the various interactions. Potential target sites are then assessed using the weighted criteria and assigned a score. Although the algorithms have limitations and do not always correctly predict insertion frequency, typically a targetron efficient enough to be screened for site-specific insertion via colony PCR without selection can be found for any given stretch of 1,000 base pairs of DNA. Off-target integrations by the Ll.LtrB and EcI5 targetrons are rare and can generally be avoided by the prudent step of scanning the genome for closely matching target sites. However, the specificity of targetrons could vary for different target sites, making it important to confirm desired single integrations by Southern hybridization.

The actual retargeting process is carried out by using PCRs that modify the EBS/δ sequences within the intron to base pair to the DNA target site and simultaneously modify the IBS sequences upstream of the intron to base pair to the retargeted EBS sequences to allow the intron to splice out of a precursor RNA [6,7]. The PCR product corresponding to a segment of the intron and upstream exon is then cloned into a targetron expression vector (see below). Alternatively, the entire region covering the IBS1 and 2 and the EBS1, 2 and δ sequences can be commercially synthesized in a single DNA molecule (for example, as a gBlock sold by IDT) that can be cloned directly into the vector [60]. The outlying δ’ or EBS3/IBS3 positions are typically adjusted by cloning the PCR product into one of four parallel targetron vectors already containing the correct bases for these interactions.

**Figure 5:**
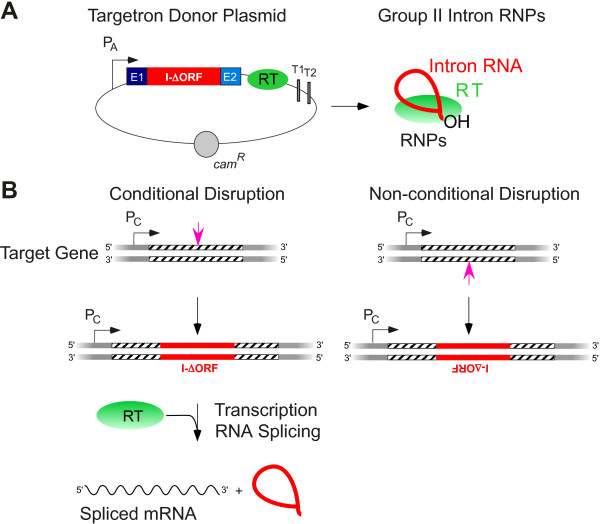
**Targetron donor plasmid and use of targetrons for conditional and non**-**conditional gene disruptions. (A)** Targetron donor plasmid. The plasmid expresses a modified group II intron with the reverse transcriptase (RT) ORF deleted (I-ΔORF) and flanked by short exons under control of an active promoter (P_A_), which can be either inducible or constitutive. The RT ORF is expressed in tandem from a location just downstream of E2. Protein-assisted splicing of the primary transcript produces a ribonucleoprotein (RNP) complex, which contains the group II intron RT bound to the excised intron lariat RNA and which promotes site-specific integration of the intron into DNA target sites via retrohoming (see Figure 3). **(B)** Use of targetrons for conditional and non-conditional gene disruptions. Conditional disruptions are obtained when the intron is targeted to insert into the top or sense strand of the target gene. Transcription of the target gene from its own promoter in the host chromosome (P_C_) results in a primary transcript from which the intron can be removed by providing the RT, which promotes protein-assisted RNA splicing. Non-conditional disruptions are obtained by targeting of the intron to the bottom or antisense strand, which results in the insertion of the intron in an antisense orientation relative to that of the target gene. Transcription of the target gene then yields a primary transcript containing the complement of the intron, which is inactive and cannot be removed by RNA splicing.

For biotechnological applications, targetrons are typically expressed from a donor plasmid that is transformed or conjugated into the desired host (Figure 5A). In *E. coli*, targetron donor plasmids have used a T7 promoter driven by T7 RNA polymerase integrated into the chromosome or expressed from a separate plasmid [6]. However, endogenous host or plasmid promoters can also be used in *E. coli* and are commonly employed for targetron expression in other bacteria [61-63]. A broad-host-range targetron expression plasmid, pBL1, uses an m-toluic acid-inducible promoter, which is not dependent upon specific host factors for induction [64]. The typical configuration for the targetron cassette is one in which the ORF encoding the RT is removed from DIV of the intron and expressed in tandem. This increases the efficiency of retrohoming and allows for disruptions of the targeted gene to be either conditional or non-conditional, depending on whether the intron is targeted to insert into the sense or antisense strand of the gene and whether or not the RT remains present to aid in splicing of the intron from the mRNA (Figure 5B) [61,62].

**Figure 6:**
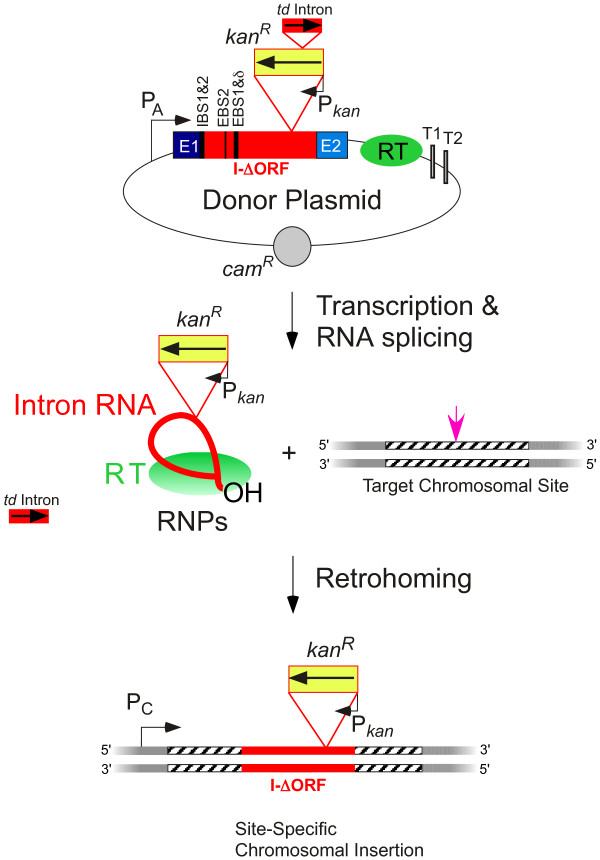
**Use of a retrotranscription**-**activated marker (RAM) to select for targetron integrations.** A targetron with a RAM cassette is expressed from a donor plasmid. The RAM cassette consists of a selectable marker gene, such as an antibiotic-resistance gene (*kan*^R^), inserted within the intron DIV in the antisense orientation, but interrupted by an efficiently self-splicing group I intron (the phage T4 *td* intron) in the sense orientation, thereby preventing expression of the *kan*^R^ marker gene from the donor plasmid. Transcription of the targetron carrying the RAM from the donor plasmid yields a primary transcript from which the group I intron is spliced to reconstitute the *kan*^R^ marker and the group II intron is spliced to yield RNPs that retrohome into a DNA target site. After retrohoming, the reconstituted *kan*^R^ marker is expressed from its own promoter, enabling selection for targetron integrations.

It is also possible to select for targetron integration by using a retrotransposition-activated marker (RAM) [59,63] (Figure 6). This involves including in the targetron a selectable marker, such as a *kan*^R^ antibiotic-resistance gene, that is inserted in the antisense orientation and is itself disrupted by a self-splicing group I intron in the sense orientation, a configuration first developed to detect rare retrotranspositions to ectopic sites [65]. The marker can only be expressed after splicing out of the group I intron and reverse transcription of the RNA intermediate into DNA, as occurs during the process of retrohoming. An Ll.LtrB targetron containing a trimethoprim-resistance-RAM (*Tp*^R^-RAM) and randomized EBS/δ sequences was used to construct an *E. coli* gene disruption library [59]. After targetron expression, Tp^R^ colonies contained targetrons inserted into different genes with complementary IBS/δ’ sequences, and these validated targetrons could be recovered by simple PCR and used to obtain the same disruption in other *E. coli* strains [66], providing an alternative to the use of a targeting algorithm.

### Targetron use in bacteria

Targetrons have been used in a wide range of bacteria, including medically and commercially important species that had been recalcitrant to gene targeting by other methods (Table 1). Compared to other bacterial gene targeting methods, advantages of targetrons are their wide host range (the Ll.LtrB targetron works in virtually all bacteria), ability to function in either RecA^+^ or RecA^-^ hosts, very high integration efficiencies (typically 1 to 100% without selection), and ease of retargeting via a computer algorithm and simple PCR reactions that are amenable to high-throughput approaches.

**Table 1:** Bacteria in which targetrons have been used successfully

**Genus**	**Primary references**
*Agrobacterium*	[64]
*Azospirillum*	[67]
*Bacillus*	[68]
*Clostridium*	[63,69]
*Ehrlichia*	[70]
*Escherichia*	[6,42]
*Francisella*	[71]
*Lactococcus*	[61]
*Listeria*	[72]
*Paenibacillus*	[73]
*Pasteurella*	[74]
*Proteus*	[75]
*Pseudomonas*	[64]
*Ralstonia*	[76]
*Salmonella*	[6]
*Shewanella*	[60]
*Shigella*	[6]
*Sinorhizobium*	[57]
*Sodalis*	[77]
*Staphylococcus*	[62]
*Vibrio*	[78]
*Yersinia*	[79]

It is relatively simple to tailor the commercially available Ll.LtrB or EcI5 targetron cassettes for use in different bacterial hosts. This typically requires re-cloning the targetron cassette from the provided donor plasmid into an established host-specific or broad-host-range expression plasmid with a strong promoter. Continuous targetron expression, which can lead to off-target integrations, can be avoided by using an inducible promoter or a donor plasmid that is readily curable in the absence of selection. A RAM capable of functioning in the desired bacteria can also be introduced into the intron, but targeting frequencies are typically high enough to screen for targetron insertions by colony PCR, making such a marker dispensable. The ClosTron, which has made possible gene targeting in a wide range of notoriously difficult *Clostridum* spp., is a highly successful example of adaptation of the Ll.LtrB targetron from a commercial kit [63,80], and similar adaptations of the Ll.LtrB targetron have been made for a variety of other bacteria (for example, [62,64,71,81]). Because the initial reverse splicing and target-DNA-primed reverse transcription reactions are catalyzed by group II intron RNPs, and because the late steps of second-strand synthesis and cDNA integration are performed by common host factors [30,31,37,82,83], there are in principle no limitations to the number of bacterial species in which targetrons might function. As mobile group II introns are present in the genomes of some archaea [84], it seems likely that targetrons will prove useful in archaea, as well.

### Applications of targetrons in bacteria

Targetrons have most frequently been used to generate knock-outs in bacteria. A great deal of work has been done using this method, with examples including identifying virulence factors [70,72,74,85-88] and potential drug targets [89,90], and examining the combinatorial effect of different genomic loci on protein expression [91].

Targetrons have been particularly widely used in strains of the genus *Clostridium*. Suicide plasmids were previously the only method of utility in these strains [63], but since Clostridia typically have very low transformations frequencies (for instance, more than a milligram of plasmid is required to transform *Clostridium acetobutylicum*[92]), suicide plasmids are difficult to use in these organisms. Targetrons have thus greatly increased our understanding of and ability to engineer Clostridia, many of which are of medical and industrial importance. For instance, Clostridia include a number of biofuel-producing strains, and targetrons have come into frequent use to aid in understanding the metabolism of these strains and to engineer them for higher yields [92-110]. Targetron-mediated knockouts have been used in a large number of studies on sporulation, germination, and other aspects of the biology of *Clostridium difficile*, a leading cause of diarrhea in hospitals [88,111-143]. Targetron technology has also benefitted the study of toxin production, sporulation, and other biological processes in *Clostridium botulinum*[144-153], *Clostridium perfringens*[69,85,154-164], and *Clostridium sordellii*[87,165]. Work on developing targetrons for the thermophilic bacterium *Clostridium thermocellum* is discussed in more detail below.

Many bacteria of interest are difficult to transform due to restriction-modification systems. In *Staphylococcus aureus*[81], *Clostridium acetobutylicum*[166], and *Clostridium cellulolyticum*[167], targetrons were used to knock out restriction enzymes, thereby opening clinical and environmental isolates to systematic mutational analysis. Besides *S. aureus* and the *Clostridium* species mentioned previously, targetrons have been developed for use in other pathogenic bacteria, such as *Francisella tularensis*[71], *Bacillus anthracis*[68,168], *Listeria monocytogenes*[72], *Pasteurella multocida*[74], *Vibrio cholerae*[78], and *Ehrlichia chaffeensis*[70], opening up the possibility of using targetrons to develop vaccine strains of these organisms.

Targetrons have also been used to deliver cargo genes, including genes for fluorescent proteins [91], phage resistance [61], and antigens for release into a host’s digestive system as a live vaccine [169]. Unstructured sequences of less than 100 nts in length can usually be carried without impacting intron mobility. Longer sequences may impair functionality, and sequences above 1,000 nts can drastically decrease efficiency. DIV, particularly the DIVb loop, has been shown to be the best location to insert cargo genes for minimal impact on intron mobility [170]. Targetrons have also been used to induce targeted genomic deletions via homologous recombination, albeit at much lower efficiencies than are possible in tandem use with recombinases [171].

Finally, the relative simplicity of targetron retargeting, combined with the falling costs of gene synthesis [172] and the increasing ability to automate the laboratory techniques involved [173,174], opens the door to high-throughput construction of targetrons for simultaneous integration into a multiplicity of loci. Applications could include rapidly generating whole-genome knock-out libraries for novel organisms and testing in parallel different combinations of mutants discovered in random screens in order to, for example, improve the yield of a target metabolite or develop a suitable vaccine strain for a pathogenic organism. Two other recent extensions of targetron technology in bacteria are discussed below.

### A thermotargetron for gene targeting in thermophiles

**Figure 7:**
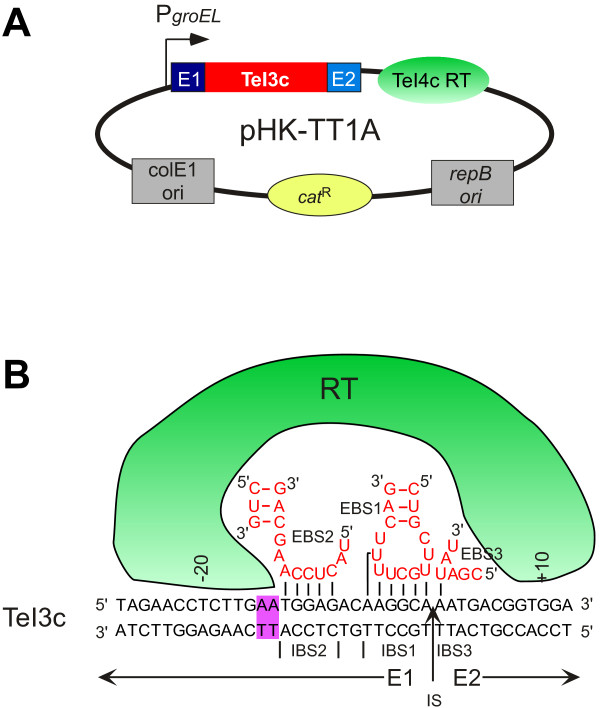
**Thermotargetron expression vector and DNA target site recognition. (A)** The thermotargetron expression vector pHK-TTA1 carries replication origins for *E. coli* (ColE1 ori) and Gram-positive bacteria (*repB* ori) and a chloramphenicol-resistance marker gene (*cat*^R^) that functions in both Gram-negative and Gram-positive bacteria. The thermotargetron cassette consisting of the upstream *Thermosynechococcus elongatus* TeI3c group IIB intron and the downstream TeI4c RT is expressed from a *Clostridium thermocellum groEL* promoter. **(B)** The figure shows DNA target site interactions for the wild-type TeI3c group IIB intron used in the thermotargetron. Portions of the intron RNA involved in EBS1-IBS1, EBS2-IBS2, and EBS3-IBS3 base-pairings interactions with the DNA target site are shown in red. Purple highlights indicate the two base-pairs in the 5’ exon (E1) that are important for DNA targeting and are likely recognized by the TeI4c reverse transcriptase (RT). IS, intron-insertion site.

Bacterial thermophiles are widely used for the production of chemicals and thermostable proteins but in many cases have inefficient transformation systems and have proven difficult to genetically engineer by conventional methods relying on homologous recombination [175-177]. Recently, a thermotargetron for gene targeting in thermophiles was developed based on a group IIB intron (denoted TeI3c) and RT (TeI4c RT) from the thermophilic cyanobacterium *Thermosynechococcus elongatus*[178] (Figure 7A; see also the diagram of the TeI4c RT in Figure 2B). Unlike other group II introns that have been made into targetrons, TeI3c is a naturally ORF-less group II intron, and TeI4c is an RT that is encoded by another group II intron but mobilizes TeI3c efficiently.

This TeI3c/4c thermotargetron was used for efficient gene targeting in *Clostridium thermocellum*, an organism used in the consolidated bioprocessing of lignocellulose biomass [178,179]. Like many species of Clostridia, *C. thermocellum* has low, variable transformation frequencies. An important feature of the thermotargetron is its high integration efficiency, 67 to 100% without selection for seven successful gene disruptions, making it possible to identify disruptants by colony PCR of only a small number of transformants. Gene disruptions that block pathways leading to by-products of cellulose degradation increased cellulolytic ethanol production in *C. thermocellum*[178].

Another notable feature of the thermotargetron is that it recognizes DNA target sites almost entirely by base pairing of the intron RNA (11-bp), while the RT recognizes only two bases (Figure 7B). The contribution of the RT to DNA melting appears to be largely dispensable at higher temperatures. This feature is advantageous because it increases the number of potential target sites and should facilitate the targeting of short ORFs and small non-coding RNAs, not only in thermophiles but also potentially in mesophiles that can tolerate short times at elevated temperatures (45 to 48°C). A downside of the more limited protein recognition, however, is that it decreases DNA target specificity, thus requiring greater attention to targetron design to avoid integration into closely matching off-target sites. The decreased target specificity may also contribute to the lower success rate for gene disruptions (7 of 25 targetrons in initial tests), which could be due in part to deleterious off-target integrations. This situation should be ameliorated by the development of algorithms to minimize off target integrations, as done for other targetrons. The TeI3c/4c thermotargetron functions in both Gram-negative and Gram-positive bacteria and should be adaptable to a wide variety of thermophiles.

### Use of targetrons for large-scale genome engineering

Targetrons have recently been adapted for carrying *lox* sites to facilitate large-scale genome engineering [60]. While recombinase sites have been previously included in targetrons, they had rarely been used for any purpose other than removing selectable markers after integration [59,80]. *Lox* sites and other recombinase recognition motifs with palindromic sequences can form stable hairpin structures upon transcription into RNA. In the absence of a selectable marker, such hairpin structures can significantly impair the functionality of both the Ll.LtrB and EcI5 targetrons. This effect was mitigated by adding non-base-pairing regions to the base of the hairpin structures, which presumably made the hairpins more flexible, such that they no longer interfered with the catalytic structures of the intron. These results point out the importance of considering structure when designing targetrons to carry cargo.

**Figure 8:**
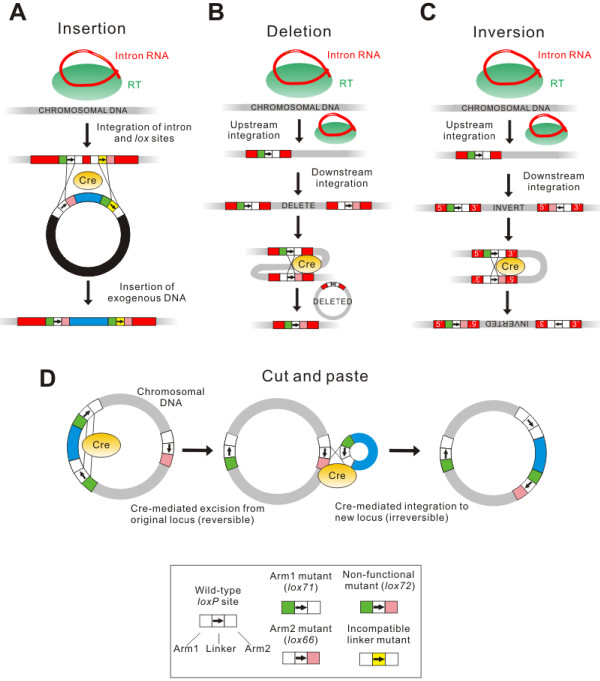
**Genome editing operations using targetrons and recombinases.** Cre/*lox* is the recombinase system used in this example. **(A)** Inserting exogenous DNA (recombinase-mediated cassette exchange). Two *lox* sites having incompatible linker regions and differing arm mutations (for example, *lox71* and *lox66*) are delivered to the genome using an intron. The sequence to be inserted is then delivered between *lox* sites identical to those in the genome except having opposite arm mutations. The formation of non-functional *lox* sites (*lox72*) makes the process irreversible. **(B)** Procedure for deleting genomic sequences. After delivery of *lox* sites (*lox71* and *lox66*) on targetrons, Cre-mediated recombination then deletes the intervening region, leaving a non-functional *lox* site (*lox72*) behind. **(C)** Procedure for inverting genomic sequences. The procedure is the same as in panel **B**, except the *lox* sites have opposing orientations. **(D)** Procedure for one-step cut-and-paste after using introns to position *lox* sites (two *lox71* sites and one *lox66* site) as shown. The first (reversible) step is Cre-mediated deletion, followed by Cre-mediated reinsertion at the target site that is made irreversible by the formation of a non-functional *lox* site (*lox72*).

Since both targetrons and the Cre/*lox* system [180] function well in a wide variety of organisms, the combination of both allows for a powerful and generalized genome engineering system, where previously engineering solutions typically needed to be developed for each organism. Once the *lox* or other recombinase sites are positioned using the targetrons, a variety of operations are possible. Figure 8 shows schematics for using the system to engineer large-scale insertions, deletions, inversions, and one-step ‘cut-and-paste’ operations in which large DNA segments are translocated from one genomic site to another. Manipulations of any size are possible, within the constraints of gene content and genome structure. Among other manipulations, the system was used to deliver a 12-kb polyketide synthase operon to the genome of *E. coli*; move 100 kb of the *E. coli* genome to another locus 1.5 Mb away; delete a pathogenicity island from *Staphylococcus aureus*; and invert approximately one third of the *Bacillus subtilis* genome. Intragenomic recombinations mediated by Cre-*lox* occurred at essentially 100% efficiency, and intermolecular recombinations occurred at 40 to 100% efficiency, without the need to place any selectable markers in the genome. The method can be expected to function in any organism in which targetrons can be made to work.

These examples are likely but the first in a series of innovations that will allow targetrons to be used for large-scale genomic engineering. There are currently few alternatives that allow the facile, site-specific introduction of genetic material into microorganisms. While some organisms, such as *Streptococcus pneumoniae*[181] and *Acinetobacter*[182], have relatively robust systems for homologous recombination, most others do not. Similarly, while methods such as recombineering [183,184] and MAGE [174] have been developed that allow PCR products and oligonucleotides to be readily introduced into *E. coli* in a site-specific manner, these methods do not scale to most other microorganisms. Targetrons are essentially the only tool that can be used to site-specifically ‘punctuate’ the genomes of a wide array of bacteria, as has previously been observed for recalcitrant thermophilic strains and Clostridia, discussed elsewhere in this review. While *lox* sites have been introduced to promote site-specific recombination, the option also exists to introduce a wide variety of other short genetic elements that can impact phenotype, including promoters, terminators, leader sequences, affinity tags, and even origins of replication. The use of targetron libraries [59,66] to seek out sites that lead to improved functionality, combined with the use of efficient targetron insertion to rapidly introduce multiple targetrons into a single strain, either serially or in parallel, makes targetrons the tool of choice for the engineering of industrially-relevant microorganisms.

### Prospects for targetron use in eukaryotes

Although efficient eukaryotic gene targeting technologies have been developed, including Zn-finger nucleases, TALENS, and CRISPR-based systems, targetrons offer the advantages of greater ease of retargeting than Zn-finger nucleases or TALENS and potentially higher DNA target specificity than any of the other methods. However, the barriers to targetron use in eukaryotes include the requirement for delivering RNPs containing a large, structured group II intron RNA to the nucleus, as well as the relatively high Mg^2+^ concentrations required for group II intron RNA function. Group II introns evolved to function in bacteria, whose free Mg^2+^ concentrations are generally 1 to 4 mM [185], whereas in eukaryotes, Mg^2+^ concentrations are <1 mM and possibly lower in nuclei, where Mg^2+^ is sequestered by binding to large amounts of DNA [186,187]. These lower Mg^2+^ concentrations constitute a barrier to group II intron invasion of nuclear genomes and limit their efficiency for gene targeting in eukaryotes. Additional host defense and innate immunity mechanisms could also come into play.

Initial studies showed that Ll.LtrB targetron RNPs introduced into mammalian cells by transfection could integrate into separately transfected plasmid target sites albeit at low efficiency [5] and envisioned methods that might be used for targeted repair of human genes [188]. In a later systematic study testing the feasibility of using targetrons in eukaryotes, Ll.LtrB targetron RNPs were microinjected directly into *Xenopus laevis* oocyte nuclei and tested for retrohoming and gene targeting via double-strand-break-stimulated homologous recombination in plasmid assays [34]. These studies showed that retrohoming and targeting via group II intron-stimulated homologous recombination occurred efficiently (up to 38% and 4.8% of plasmid target sites, respectively), but required the injection of additional Mg^2+^, sufficient to obtain an intracellular concentration of 5 to 10 mM. A similar requirement for the injection of additional Mg^2+^ for retrohoming was found for targetron RNPs injected into *Drosophila* and zebrafish embryos [34]. Injection of targetron RNPs plus Mg^2+^ have given targeted integration into the chromosomal yellow gene in flies at frequencies up to 0.12% in pooled embryos and 0.021% in pooled adult files [189], and in *X. laevis*, a different approach, using group II intron RNPs for site-specific DNA modification in sperm nuclei followed by *in vitro* fertilization, gave targeted integration at frequencies sufficiently high to detect knockouts in a single copy *mitF* gene by PCR screening of tail clippings (M Mastroianni, J Yao, and AM Lambowitz, unpublished data). However, the frequencies were variable and further improvements in efficiency and reliability are needed for these to become routine methods.

There is some prospect that more active group II introns with enhanced retrohoming in eukaryotes can be selected by directed evolution approaches. Recent work showed that Ll.LtrB introns that retrohome more efficiently at lower Mg^2+^ could be selected in an *E. coli* mutant deficient in Mg^2+^-transport [190], laying the groundwork for direct selections of group II introns that function more efficiently in eukaryotic cells. The recent group II intron RNA X-ray crystal structures [19-21] may also enable rational design approaches to enhancing group II intron function. If these efforts prove successful, the same rationales that are driving the use of targetrons for genomic engineering in bacteria will extend to genomic engineering in eukaryotes.

### Thermostable group II intron reverse transcriptases

Reverse transcriptases are widely used in biotechnology for applications involving cDNA synthesis, such as qRT-PCR and RNA-seq. Most if not all of these applications would benefit from using RTs that synthesize cDNAs with high processivity and fidelity. However, the retroviral RTs that are commonly used for these methods have inherently low fidelity and processivity, reflecting that these enzymes evolved to help retroviruses evade host defenses by introducing sequence variations and rapidly propagating successful ones by RNA recombination [191]. Vast sums have been expended to engineer variants of retroviral RTs that overcome these inherent deficiencies.

By contrast, group II intron RTs evolved to have high processivity and fidelity, reflecting their function in retrohoming, which requires synthesis of an accurate, full-length cDNA copy of a highly structured group II intron RNA [8,9]. Other advantageous characteristics of group II intron RTs are their lack of RNase H activity, which enables reuse of RNA templates, and their difficulty in initiating on DNA templates, which preserves RNA strand information by minimizing recopying of cDNAs [23,31].

**Figure 9:**
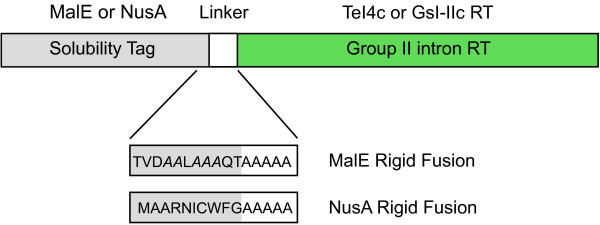
**Thermostable group II intron reverse transcriptase (RT) fusion proteins.** High-level expression of thermostable group II intron RTs that remain soluble when purified free of bound RNAs is achieved by expressing the RT as a fusion protein with a solubility tag, such as MalE or NusA, linked to the N-terminus of the RT via a non-cleavable rigid linker [10]. In these examples, the rigid linker consists of five alanine residues and the MalE and NusA tags are modified (MalE, charged amino acids changed to alanines (italics); NusA, two C-terminal amino acids deleted) to reduce conformational flexibility at the fusion junction and achieve an optimal spacing between the solubility tag and the group II intron RT [10].

In a recent technical advance that makes group II intron RTs available for widespread use as tools for research and biotechnology, general methods were developed that enable their high-level expression in bacteria and their purification in active form free of tightly bound RNA [10]. These methods involve the expression of group II intron RTs as fusion proteins with a solubility tag, such as MalE or NusA, attached to the N-terminus of the RT via a non-cleavable rigid linker (Figure 9). The attached solubility tag enables the protein to remain soluble when freed of the intron RNA, and the rigid linker minimizes interference of the tag with RT function.

By applying the above methods to group II intron RTs from the bacterial thermophiles *Thermosynechococcus elongatus* and *Geobacillus stearothermophilus* (Figure 2B), it was possible to obtain thermostable group II intron RT fusion proteins that synthesize cDNAs at temperatures up to 81°C with much higher processivity and two- to four-fold higher fidelity than retroviral RTs [10]. The high processivity of group II intron RTs is advantageous for synthesizing long cDNAs that preserve information about alternatively spliced RNAs and for RNA footprinting and structure mapping using RNA modification reagents, where premature terminations by retroviral RTs at unmodified sites result in high background and loss of information. The higher fidelity of group II intron RT should benefit applications, such as tumor profiling, that require the analysis of sequence variants.

We are still at the early stages of developing methods and applications utilizing the novel properties of these enzymes. Group II intron RTs behave differently from retroviral RTs, both in terms of optimal conditions for different applications and their tighter binding to nucleic acids, which necessitates different types of clean-up procedures for cDNA products. Consequently, group II intron RTs cannot simply be substituted into protocols developed for retroviral RTs and must be optimized for each application. In a published application, a thermostable group II intron RT was used to generate RNA-seq libraries of human mRNAs, using an oligo(dT)_42_ primer [10]. The resulting libraries showed relatively uniform 5’ to 3’ coverage of all size classes of human mRNAs, including those >7 kb, whereas parallel libraries prepared using the thermostable retroviral RT, SuperScript III, showed a strong bias for reads near the 3’ ends of mRNAs, reflecting premature terminations. The ability to obtain RNA-seq libraries with uniform 5’ to 3’ coverage using an oligo(dT) primer avoids steps such as ribodepletion and RNA fragmentation, which are needed to minimize rRNA contamination and obtain uniform coverage in libraries prepared using retroviral RTs. The minimal manipulation needed to prepare whole cell RNA-seq libraries using group II intron RTs may be useful for procedures that start with small amounts of RNA, such as transcriptome analysis from single cells.

**Figure 10:**
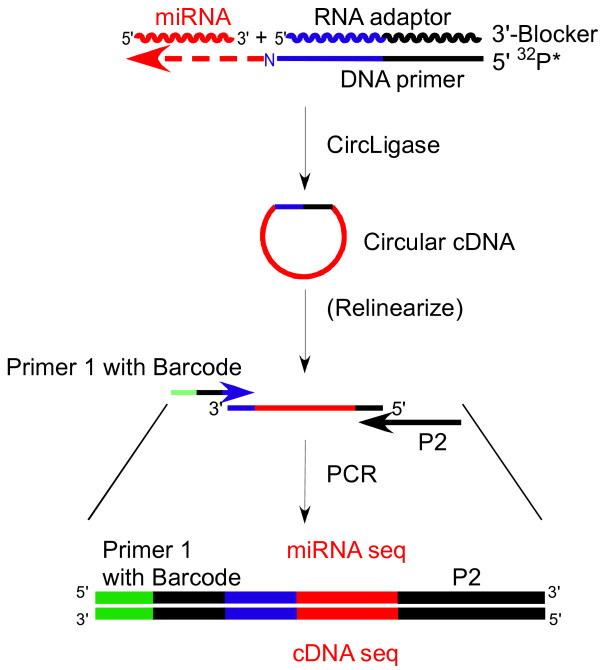
**Thermostable group II intron reverse transcriptase (RT) template switching method for attachment of adaptor sequences for RNA**-**seq.** The RT binds to an initial substrate consisting of a synthetic RNA oligonucleotide adaptor and an annealed DNA primer, with the RNA oligonucleotide having a blocking group attached to its 3’ end to impede recopying by the RT. In the example shown, the initial substrate has a single nucleotide 3’ overhang of the DNA primer (denoted N to signify that this nucleotide can be A, C, G, or T or a mixture of all four nucleotides). The single nucleotide 3’ overhang can facilitate template switching by base pairing to a complementary 3’ terminal nucleotide of a target RNA, which is a miRNA in the example shown. After template switching, the RT copies the target miRNA. The resulting cDNA containing the adaptor sequence seamlessly linked to the miRNA sequence is circularized with CircLigase and amplified by PCR with Primers P1 and P2 that introduce primer-binding sites and barcodes for RNA-seq. Relinearization of the circularized cDNA, which can increase the efficiency of the PCR in some cases, is an optional step [10].

In addition to their higher processivity and fidelity than retroviral RTs, group II intron RTs have a novel end-to-end template switching activity in which the RT synthesizes a cDNA copy of one template and then switches directly to the 3’ end of a second template [10]. As illustrated in Figure 10, this template-switching activity can be used to seamlessly link RNA-seq adaptor sequences containing primer-binding sites and barcodes to cDNAs during reverse transcription, thereby avoiding an additional inefficient and bias-inducing step of using RNA ligase for linker ligation. In the example shown, the group II intron RT initiates from a short synthetic RNA oligonucleotide adaptor with an annealed DNA primer. It then switches to the 3’ end of a target miRNA, yielding a continuous cDNA containing the RNA-seq adaptor sequence seamlessly linked to the miRNA sequence.

Like other DNA and RNA polymerases, group II intron RTs are prone to add extra non-templated nucleotides to DNA upon reaching the end of an RNA template (ref. [10] and references therein), which could lead to non-seamless junctions and biases during template switching. This problem is avoided by using an initial template/primer substrate consisting of a synthetic RNA oligonucleotide annealed to a DNA primer that leaves a single nucleotide 3’ overhang. This 3’ overhang nucleotide base pairs with the 3’ terminal nucleotide of the second RNA template, resulting in a seamless switch to the second template. A specific 3’ overhang nucleotide can be used to direct the RT to a specific target RNA, while a mixture of 3’ overhang nucleotides is used to minimize bias for mixtures of templates having different 3’ RNA ends.

After template switching, the resulting cDNA linked to adapter sequences is circularized with CircLigase and PCR amplified to generate an RNA-seq library (Figure 10). By incorporating an additional step for removal of a 3’ phosphate of the target RNAs, the method can also be applied to protein- and ribosome-bound RNA fragments in procedures such as HITS-CLIP, CRAC, RIP-Seq, and ribosome profiling. Because thermostable group II intron RTs can template-switch to RNAs with modified 3’ ends and reverse transcribe through highly structured RNAs, the method can potentially lead to the identification of novel miRNAs and other non-coding RNAs that cannot be cloned by current methods using retroviral RTs.

## Conclusions

The biotechnological applications of mobile group II introns and their RTs are an example of how basic research into biochemical mechanisms and evolution can lead to unexpected practical applications. Targetrons, which arose from studies of the mechanism of group II intron mobility, now provide a broad-host-range solution to knock-outs and, when combined with other technologies, such as site-specific recombinases, can be employed to make a wide variety of changes in almost any bacteria, including previously recalcitrant medically and industrially important species. Together with the prospect of targetron-mediated mutagenesis in archaea and the possibility of adapting targetrons for use in eukaryotes, targetrons are well-positioned to play an expanding role in the analysis and engineering of genomes for biotechnological and medical applications. The unique properties of group II RTs, enzymes that were discovered solely as a consequence of basic research, may ameliorate many of the problems of current *in vitro* methodologies for RNA analysis, qRT-PCR, and RNA-seq, with potentially wide applications in biomedical research, diagnostics, and biotechnology.

## Abbreviations

CRAC: Cross-linking and analysis of cDNA; DI-DVI: Group II intron RNA domains I-VI; D: DNA-binding domain of group II intron reverse transcriptases; E1 and E2: 5’ and 3’ exons; EBS: Exon-binding site; En: DNA endonuclease domain of group II intron reverse transcriptases; HITS-CLIP: High-throughput sequencing by cross-linking immunoprecipitation; IBS: Intron-binding site; I-ΔORF: Group II intron with ORF encoding the RT deleted; IEP: Intron-encoded protein; PAM: Protospacer adjacent motif; RAM: Retrotransposition-activated marker; RIP-Seq: RNA immunoprecipitation and sequencing; RNP: Ribonucleoprotein; RT: Reverse transcriptase; Tp: Trimethoprim.

## Competing interests

Targetron technology and thermostable group II intron reverse transcriptases and methods for their use are subject to issued U.S. and international patents and patent applications that are licensed by the Ohio State University and the University of Texas to InGex, LLC, which sublicenses these technologies to others for commercial applications. GM, AML, the Ohio State University, and the University of Texas are minority equity holders in InGex, LLC, and AML serves as an advisor to InGex LLC. GM and AML receive royalties for commercial use of the technologies.

## Authors’ contributions

PJE, GM, ADE, and AML all contributed to the writing of this manuscript. All authors read and approved the final manuscript.
